# Residual kinematic deviations of the shoulder during humeral elevation after conservative treatment for mid-shaft clavicle fractures

**DOI:** 10.3389/fbioe.2024.1413679

**Published:** 2024-08-09

**Authors:** Li-Wei Hung, Hsuan-Yu Lu, Tsan-Yang Chen, Ting-Ming Wang, Tung-Wu Lu

**Affiliations:** ^1^ Department of Biomedical Engineering, National Taiwan University, Taipei, Taiwan; ^2^ Department of Orthopedic Surgery, Shin Kong Wu Ho-Su Memorial Hospital, Taipei, Taiwan; ^3^ School of Medicine, College of Medicine, Fu Jen Catholic University, New Taipei City, Taiwan; ^4^ Department of Sports Management, Chia Nan University of Pharmacy and Science, Tainan, Taiwan; ^5^ Department of Orthopedic Surgery, School of Medicine, National Taiwan University, Taipei, Taiwan; ^6^ Department of Orthopedic Surgery, National Taiwan University Hospital, Taipei, Taiwan; ^7^ Health Science and Wellness Research Center, National Taiwan University, Taipei, Taiwan

**Keywords:** motion analysis, range of motion, scapula, clavicle fracture, conservative treatment

## Abstract

Despite residual functional deficits clinically observed in conservatively treated mid-shaft clavicle fractures, no study has reported a quantitative assessment of the treatment effects on the kinematics of the shoulder complex during functional movement. Using computerised motion analysis, the current study quantified the 3D residual kinematic deviations or strategies of the shoulder complex bones during multi-plane elevations in fifteen patients with conservatively treated mid-shaft clavicle fractures and fifteen healthy controls. Despite residual clavicular malunion, the patients recovered normal shoulder kinematics for arm elevations up to 60° in all three tested planes. For elevations beyond 60°, normal clavicle kinematics but significantly increased scapular posterior tilt relative to the trunk was observed in the patient group, leading to significantly increased clavicular protraction and posterior tilt relative to the scapula (i.e., AC joint). Slightly different changes were found in the sagittal plane, showing additional changes of increased scapular upward rotations at 90° and 120° elevations. Similar kinematic changes were also found on the unaffected side, indicating a trend of symmetrical bilateral adaptation. The current results suggest that shoulder kinematics in multi-plane arm elevations should be monitored for any compromised integrated motions of the individual bones following conservative treatment. Rehabilitation strategies, including muscle strengthening and synergy stability training, should also consider compensatory kinematic changes on the unaffected side to improve the bilateral movement control of the shoulder complex during humeral elevation.

## Introduction

Clavicle fractures account for 35%–44% of injuries to the shoulder girdle and 2.6%–10% of all adult fractures (Nordqvist and Petersson, 1994; Postacchini et al., 2002), with an annual incidence ranging from 0.29 to 0.64 per 1,000 adults (Khan et al., 2009; Hsiao et al., 2012). The risk is higher in males under 30 and adults over 70 years of age (Court-Brown and Caesar, 2006). Typically, clavicle fractures are classified into three groups based on the anatomical site of the fracture, mid-shaft clavicle fractures being the most frequently occurring group (Allman, 1967). If not appropriately managed, clavicle fractures can result in short-term disability and pain, eventually causing long-term deformity and disability (Lenza et al., 2019).

Conservative treatment, e.g., a simple sling or figure-eight bandage, has traditionally been used to manage mid-shaft clavicle fractures with a good prognosis and low risk of nonunion (Neer, 1960; Rowe, 1968). However, primary surgical treatment may be a better option for a subgroup of more severe fractures to lower the incidence of symptomatic malunion or nonunion (Bernstein, 2007). The nonunion rate for displaced fractures treated non-surgically was higher, and the time to return to activities was longer than those treated surgically (Hill et al., 1997; Morgan et al., 2010; Murray et al., 2013). Due to the rigid design and the ability to recover rotational deformity, surgeons prefer treatment with a locking plate for displaced clavicle fractures, and this has become the current standard (Ferran et al., 2010; Ban et al., 2016). Yet, despite the use of stable implants, the shoulder complex has also been documented to have poor functional results, primarily due to scapular dyskinesis (Mirzatolooei, 2011; Robinson et al., 2013; Shields et al., 2015). Therefore, while severely displaced or comminuted fractures may require surgical fixation, non-surgical conservative treatment remains a popular choice for less severe clavicle fractures (Heuer et al., 2014).

The normal function of the shoulder complex relies on the coordinated movement of the clavicle, scapula, humerus, and thorax, interacting through joints such as the acromioclavicular and sternoclavicular joints. The clavicle acts as a structural support and a link to the shoulder mechanism, maintaining the scapula’s position and enabling a wide range of shoulder motions. The scapula provides muscle attachment sites and contributes to glenohumeral joint stability and coordinated movements of the shoulder mechanism. A mid-shaft clavicle fracture can disrupt these functions, leading to scapular dyskinesia, a condition characterised by an abnormal motion or positioning of the scapula. Malunion of mid-shaft clavicle fractures is also associated with scapular dyskinesis (Hillen et al., 2010; Kim et al., 2019). Thus, measuring the scapular kinematics helps assess the recovery of the shoulder function following mid-shaft clavicle fractures. When treated by conservative methods, the gap of a mid-shaft clavicle fracture frequently widens over time as the sternocleidomastoid pulls the medial bone segment superiorly and the pectoralis major pulls the lateral bone segment inferiorly and medially (Mark and Lazarus, 2006). The resulting clavicle deformity may cause scapular dyskinesis after the union. Scapular dyskinesis is associated with a higher risk of long-term shoulder impairment and unsatisfactory clinical outcomes (Eskola et al., 1986; Mark and Lazarus, 2006; Postacchini et al., 2010). The scapula may be more anteriorly inclined and internally rotated (protracted) if the clavicle has a shortening deformity (Ledger et al., 2005; Kibler and Sciascia, 2010), resulting in rotator cuff related shoulder pain and subsequent rotator cuff tear (Ludewig and Cook, 2000). In scapular dyskinesis, altered scapular alignment and movements and, thus, modified lines-of-action and tension of the muscles may lead to reduced muscular efficiency and shoulder function (Eskola et al., 1986; Hill et al., 1997; Ledger et al., 2005; Myers et al., 2005; Mirzatolooei, 2011). Despite clinical observations of complications in conservatively treated mid-shaft clavicle fractures, no study, to the authors’ knowledge, has reported a kinematic assessment of the individual bones of the shoulder complex, particularly the scapula, and their interactions during functional arm elevations following such treatment.

Technically, measuring the motions of the shoulder bones during functional activities is difficult due to the extensive movement of the scapula beneath the skin and the small tubular shape of the clavicle. Previous studies mainly used medical imaging methods to evaluate the effects of clavicle malunion or shortening on the shoulder bone alignment at limited standard postures (Hill et al., 1997; McKee et al., 2003; Ledger et al., 2005; Lazarides and Zafiropoulos, 2006; Mirzatolooei, 2011). For 3D scapular motions during various arm movements, magnetic tracking sensors and inertial measurement units (IMU) have been proposed (e.g., (Lukasiewicz et al., 1999; Borstad and Ludewig, 2002; McClure et al., 2004; Rundquist and Ludewig, 2004; Cutti et al., 2008; Parel et al., 2012; Cutti et al., 2014), but both are subject to soft tissue artefacts and the encumbrance of multiple sensors or wires (Forner-Cordero et al., 2008; Weenk et al., 2013; Teufl et al., 2019).

Skin marker-based stereophotogrammetry is free from wires and widely available for 3D measurement of human movement (Cutti et al., 2005). It is also subject to skin movement artefacts like other skin-mounted sensors, so the scapular and clavicular motions are often ignored or simplified when measuring and describing the shoulder kinematics by those of the humerus relative to the trunk (Cutti et al., 2005; Garofalo et al., 2009). Indirect methods, e.g., regression equations, have also been proposed to predict the 3D scapular pose, but the accuracy was limited by the datasets used (Wu et al., 2005; Kontaxis et al., 2009; de Vries et al., 2010). For fast scapular pose measurements, a three-pointed scapular locator to be applied over the three bony landmarks was proposed with high accuracy and test-retest reliability at static positions (Kuo et al., 2018). This locator has been used to measure the shoulder kinematic changes in patients treated with internal fixation for mid-shaft clavicle fractures (Hung et al., 2021). The scapular locator approach will help measure the shoulder kinematics in patients with conservatively treated mid-shaft clavicle fractures.

The current study aimed to quantify the 3D residual kinematic deviations or strategies of the bones of the shoulder complex during multi-plane elevations following conservative treatment of mid-shaft clavicle fractures by comparing between patients with conservatively treated mid-shaft clavicle fractures and healthy controls using computerised motion analysis with a three-pointed scapular locator. The data revealed the underlying mechanisms responsible for the compromised functional performance of the shoulder complex in these patients at the level of the individual bones during standardised elevation movements.

## Materials and methods

### Subjects

**FIGURE 1:**
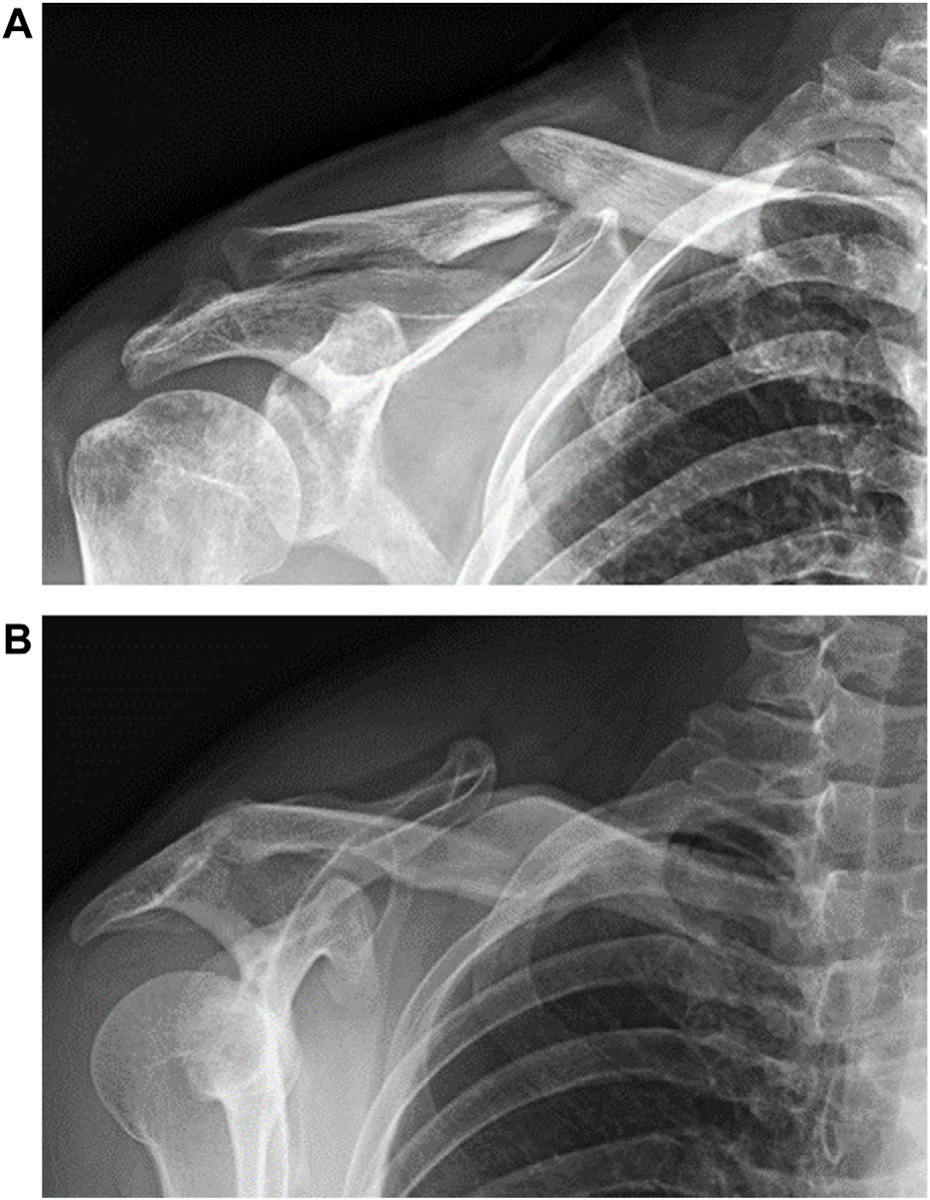
Radiographs of a typical mid-shaft clavicle fracture **(A)** before and **(B)** after treatment with a sling and a figure-of-eight bandage.

Fifteen patients with conservatively treated mid-shaft clavicle fractures (Patient group; six females and nine males; age: 31.2 ± 15 years; BMI: 23.6 ± 3.3 kg/m^2^) and 15 healthy adults (Control group; six females and nine males; age: 23.3 ± 2 years; BMI: 24 ± 2 kg/m^2^) participated in the current study with informed written consent as approved by the Institutional Review Board. Patients were eligible if they were aged between 18 and 65 years, had been treated conservatively for at least 4 weeks with a broad arm sling and a figure-of-eight bandage, and had complete fracture union 6 months after the fracture (Figure 1). Healthy adults were eligible for the Control group if they were between 18 and 65 years of age and free from existing or previous trauma to either shoulder. Participants were excluded if they had any of the following conditions: 1) open or pathological fractures, 2) displaced mid-shaft clavicle fractures that had not healed after 6 months, 3) upper limb injuries other than mid-shaft clavicle fractures, and 4) impaired neurological function, multiple traumas, or injuries related to the acromioclavicular or sternoclavicular joints. For all the patients, two senior orthopaedic surgeons (LWH and TMW) reviewed plain radiographs of the injured shoulders. The diagnosis of mid-shaft clavicle fracture and union was made by consensus. Sonography confirmed that the rotator cuff was intact without significant tenosynovitis of the biceps long head. The patients were treated with a broad arm sling with a figure-of-eight bandage for at least 3 months and had complete fracture union at 6 months post-fracture. After wearing an arm sling for 3 months, the patient began rehabilitating the affected side at the physical therapy department following an 8-week protocol (Wang and Trudelle-Jackson, 2006; Cheema et al., 2021). The patients began range-of-motion pendulum exercises as soon as the pain permitted, progressing to active range-of-motion and strengthening exercises from the fourth week. According to the patient’s condition, the physical therapist determined the immobilisation duration and the dosage of functional activities (Table 1). No patient had a floating shoulder injury (ipsilaterally displaced fractures of the glenoid or scapula and the clavicle).

**TABLE 1:** Means (standard deviations) of the demographic characteristics and clinical assessment of the affected and unaffected shoulders of the patients with mid-shaft clavicle fracture and healthy controls.

Parameters	Control group	Patient group		Ps	Pg	
		Affected	Unaffected		Affected	Unaffected
Gender (F/M)	6/9	6/9				
Age (years)	23.3 (2)	31.2 (15)			0.12	
BMI (kg/m2)	24.0 (2)	23.6 (3.3)			0.73	
DASH		7.23 (6.36)				
CMS		96.28 (5.18)				
VAS		0.7 (0.05)				
ASES		93.84 (6.87)				
Immobilisation duration (month)		4.3 (1.3)				
clavicle lengths		161.9 (21.9)	165.8 (25.0)	0.08		
Maximum arm elevation (degree)	155.4 (5.4)	144.2 (10.4)	148.5 (9.5)	0.13	0.01*	0.03*

DASH: Disabilities of Arm, Shoulder and Hand; CMS: Constant-Murley Shoulder score; VAS: Visual Analogue Scale pain score; ASES: American Shoulder and Elbow Surgeons score; Pg: p-values for comparisons between patient and Control group using independent t-test; Ps: p-values for between-limb comparisons in the patient group using paired t-test; *: Significant difference (*p* <0.05).

### Experimental protocol

This study utilised a cross-sectional case-control design. Each patient underwent computed tomography (CT) scan of both shoulders following fracture union. The CT data were then used to reconstruct the three-dimensional (3D) bone models to measure the length, percentage of shortening, and degree of angulation deformity of the clavicle. Subjective outcomes, including the Constant-Murley Shoulder (CMS) score (Constant and Murley, 1987), the Visual Analogue Scale (VAS) pain score, the American Shoulder and Elbow Surgeons (ASES) score (Richards et al., 1994) and the Disabilities of Arm, Shoulder and Hand (DASH) score (Gummesson et al., 2003), were measured at the time of the experiment.

**FIGURE 2:**
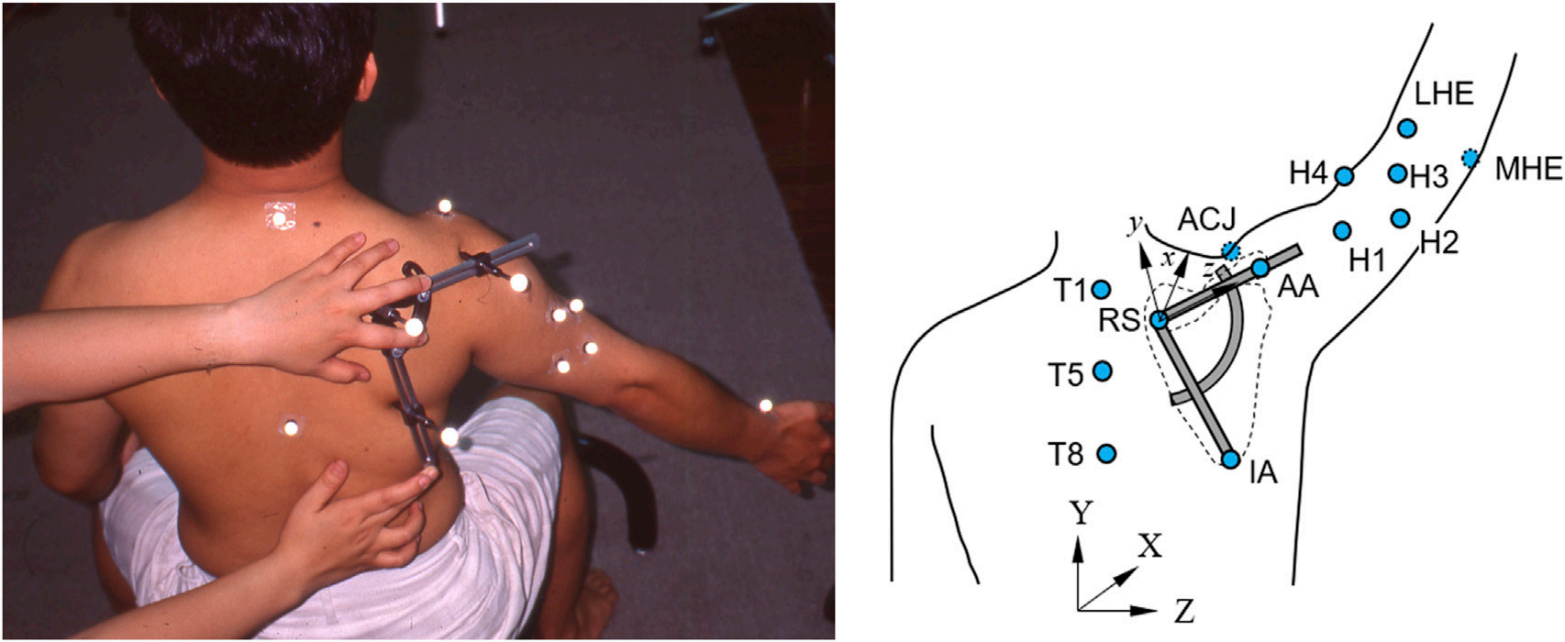
Left: Photograph showing a participant with his arm elevated at 60 degrees in the scapular plane while the examiner applied the scapular locator to measure the scapular kinematics, and Right: Schematic diagram showing the measurement of the scapular kinematics during frontal-plane arm elevation using marker-based stereophotogrammetry. The bony landmarks of the trunk, namely, the spinal process of the 1st (T1), the 5th (T5), and the 8th thoracic vertebra (T8), were each identified by an infrared retro-reflective marker. The pose of the humerus was defined by the medial and lateral humeral epicondyles (MHE and LHE) and four additional technical markers (H1-H4). The scapular pose was identified by the scapular locator, with the palpation rods adjusted to fit over the root of the scapular spine (RS), the acromial angle (AA), and the inferior angle (IA) of the scapula.

In a motion analysis laboratory, kinematics of the affected and unaffected shoulder complex during arm elevation was measured separately using skin-marker-based stereophotogrammetry. During the experiment, the subject sat on a chair with the trunk kept vertical and stationary by a support attached to the chair on the contralateral side (Kuo et al., 2018). To ensure that the trunk remained vertical and stationary during data collection, the subject was seated on a chair with the contralateral upper arm against a support attached to the chair (Kuo et al., 2018). Six infrared retro-reflective markers were placed on the xiphoid, sternal notch, mid-body of the sternum, and the spinal processes of the T1, T5, and T8 thoracic vertebrae to determine trunk posture (Figure 2). The pose of the scapula was measured using a scapular locator with three spherical-ended rods, the relative positions of which were determined by their positions on two arms rotating about a hinge joint (Figure 2) (Kuo et al., 2018). During the subject calibration at the neutral position (0° of arm elevation), the root of the spine (RS), the inferior angle (IA) and the acromion angle (AA) of the right shoulder were identified via palpation, and the experimenter adjusted the scapular locator’s palpation rods to fit over the landmarks. Firstly, the palpation rod coinciding with the axis of the locator’s hinge joint was first pointed to the RS of the scapula. Secondly, the second palpation rod sliding along one of the locator’s arms was pointed to the AA. Finally, the position of the third palpation rod was determined by sliding along the other arm and adjusting the angle between the two arms to fit over the IA (Kuo et al., 2018). Once the subject calibration was completed, the shape of the scapular locator remained fixed throughout the experiment for the subject, so the positions of the RS, AA, and IA could be measured by single retro-reflective markers attached to the top of each palpation rod. The pose of the humerus was measured using four skin markers (i.e., H1-H4), while that of the clavicle was measured using two markers placed on the acromial and sternal ends.

During data collection, each subject performed six trials of arm elevations at self-selected speed in random order on each of the sagittal, frontal, and scapular planes. They paused at specific angles (i.e., 0°, 30°, 60°, 90°, and 120°) to allow for kinematic measurements using the scapular locator. At each elevation position, a goniometer was used to confirm that the upper limb was positioned at the desired elevation angle. The experimenter then palpated the AA and RS, positioned the locator over the two landmarks, and rotated the locator to make contact with the IA with the third rod. Once the locator was adequately positioned on the scapula, the motion capture system (Vicon 512, Oxford Metrics Group, United Kingdom) measured the 3D coordinates of the locator’s markers and those on the trunk, clavicle, and humerus at 120 Hz. Complete marker data were collected for each arm elevation position, each elevation plane, each shoulder and each subject.

### Data analysis

The measured marker coordinates were used to determine the motions of the shoulder bones. The trunk, clavicle, scapula and humerus were each attached with a Cartesian coordinate system, with the positive *x*-axis pointing anteriorly, the positive *y*-axis pointing superiorly, and the positive *z*-axis pointing to the right (Wu et al., 2005). The upper arm rotational movement was described by the relative rotations between the humeral and trunk coordinate systems following a y-z-y sequence (Cole et al., 1993), corresponding to the plane of elevation, amount of elevation and internal/external rotation. Angular positions of the scapula were described relative to the trunk and calculated using a y-x-z sequence (Cole et al., 1993). The angles of the acromioclavicular (AC) joint were calculated from the rotation matrix of the clavicle bone relative to the scapula bone using a y–x–z sequence, giving protraction/retraction, upward/downward rotation, and posterior/anterior tilt, respectively. The values of the scapular and clavicular angles were then obtained relative to their neutral positions (i.e., 0° of arm elevation) for subsequent statistical analysis.

### Statistical analysis

For statistical analysis, the values of the humerus, scapula, and clavicle angles relative to the trunk and the acromioclavicular (AC) joint were obtained for each arm elevation position, each plane, each shoulder, and each subject. For each healthy subject, the corresponding values were the average data from both shoulders. Comparisons of variables between the Control group and the affected and unaffected side of the patient group were performed using a mixed-model repeated measures one-way analysis of variance (ANOVA). Once a significant group effect was found, *post hoc* tests were performed to compare the patient and Control groups using an independent t-test (Affected vs. Control and Unaffected vs. Control), while paired t-tests were used to compare the affected and unaffected sides of the patient group (Affected vs. Unaffected). SPSS version 20 was used to conduct all statistical analyses (SPSS Inc., Chicago, IL, United States), and the significance level was set at 0.05 for all tests.

### Sample size

Based on pilot results, an *a priori* power analysis using G*POWER (Erdfelder et al., 1996) was conducted for a two-group independent sample t-test comparing the shoulder bone orientations. The analysis results determined that five subjects per group would yield a power of 0.8 and a large effect size (Cohen’s d = 0.35) at a significance level of 0.05. Thus, 12 subjects for each group were sufficient for the primary objectives.

## Results

At the time of the motion trials, the subjective outcome scores for the Patient group were from good to excellent (DASH: good; Constant: very good; VAS: no pain; and ASES: no pain and excellent function, Table 1). In the Patient group, there were no significant between-side differences in maximum arm elevations (affected: 144.2° (10.4°); unaffected: 148.5° (9.5°); *p* = 0.13). Compared to the Control group, smaller maximum arm elevations (range of motion) were found in both the affected and unaffected side of the Patient group (Control: 155.4° (5.4°); affected: *p* = 0.01; unaffected: *p* = 0.03). No significant bilateral differences in the Patient group were found in the clavicle lengths (affected: 161.9 (21.9) mm; unaffected: 165.8 (25.0) mm; *p* = 0.08).

**FIGURE 3:**
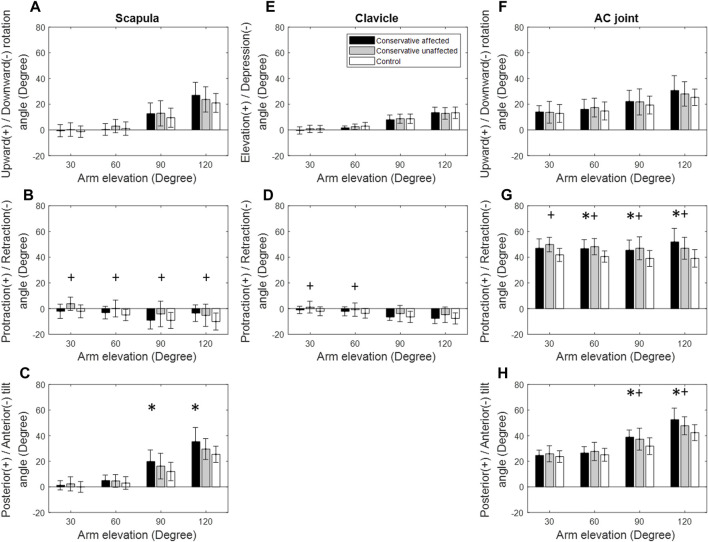
Mean rotational angles of the scapula **(A–C)**, clavicle **(D,E)**, and the acromioclavicular (AC) joint **(F–H)** during frontal-plane arm elevation for both the patient (black bars: affected side; grey bars: unaffected side) and control (white bars) groups. Significant differences between the affected side and healthy control are indicated by the symbol *, while significant differences between the unaffected side and healthy control are indicated by the symbol +. Standard deviations are shown as error bars.

Significant changes in some of the kinematic components were found on the affected side of the Patient group compared to the Control. During frontal-plane arm elevation, the Patient group demonstrated significantly increased scapular posterior tilt at 90° and 120° of elevation (Figure 3). For the clavicular orientations relative to the scapula (AC joint), the Patient group exhibited significantly increased protraction at 60°–120° of elevation and posterior tilt at 90° and 120° of elevation (Figures 3, 6). Similar findings were also found for the unaffected side (Figure 3).

**FIGURE 4:**
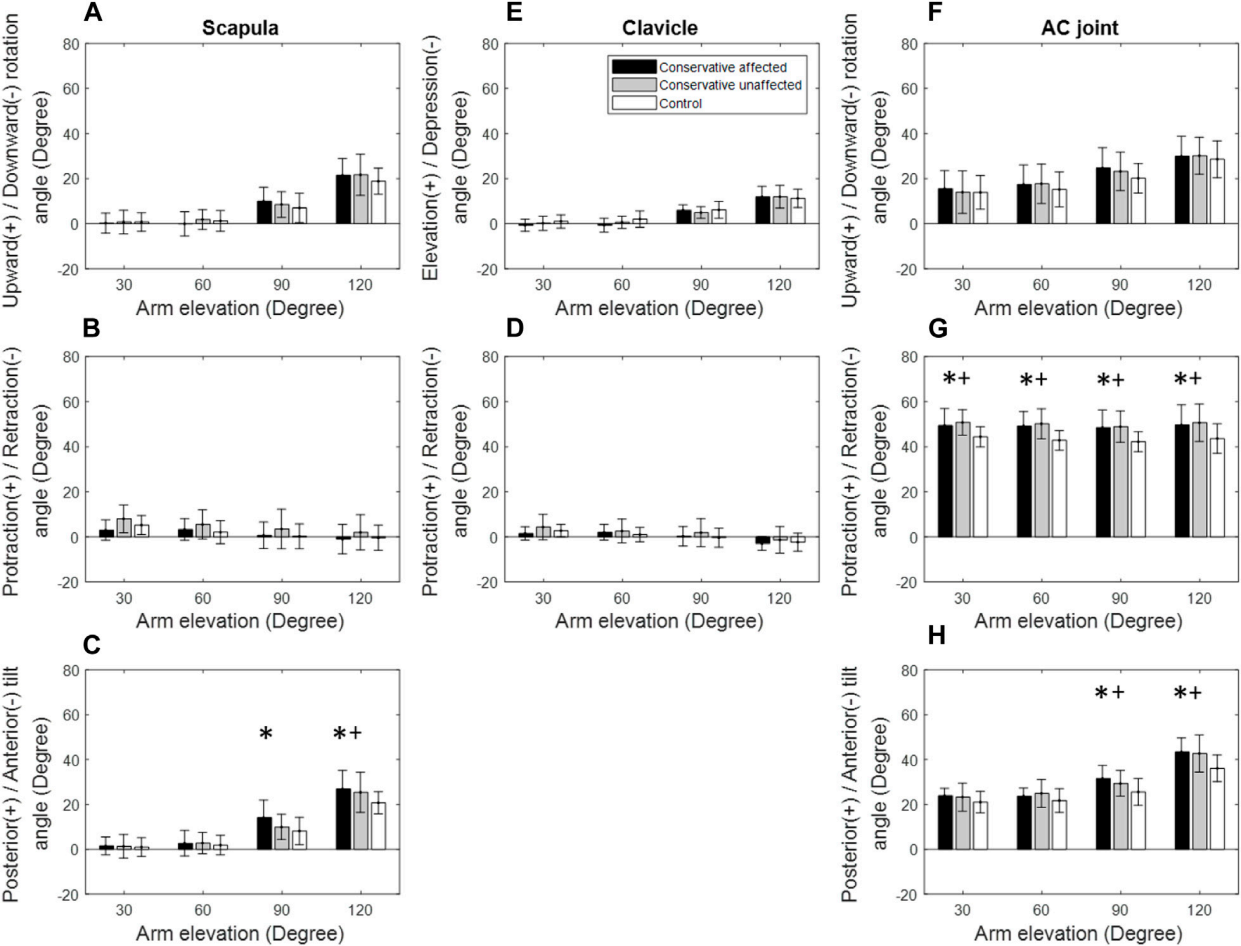
Mean rotational angles of the scapula **(A–C)**, clavicle **(D,E)**, and the acromioclavicular (AC) joint **(F–H)** during scapular-plane arm elevation for both the patient (black bars: affected side; grey bars: unaffected side) and control (white bars) groups. Significant differences between the affected side and healthy control are indicated by the symbol *, while significant differences between the unaffected side and healthy control are indicated by the symbol +. Standard deviations are shown as error bars.

During scapular-plane arm elevation, the Patient group showed significantly increased scapular posterior tilt at 90° and 120° of elevation compared to the Control, similar to those found during frontal-plane arm elevations (Figure 4). For the AC joint, the Patient group demonstrated significantly increased protraction at 30°–120° of elevation and posterior tilt at 90°–120° of elevation (Figure 4). Similar findings were found for the unaffected side (Figures 4, 6).

**FIGURE 5:**
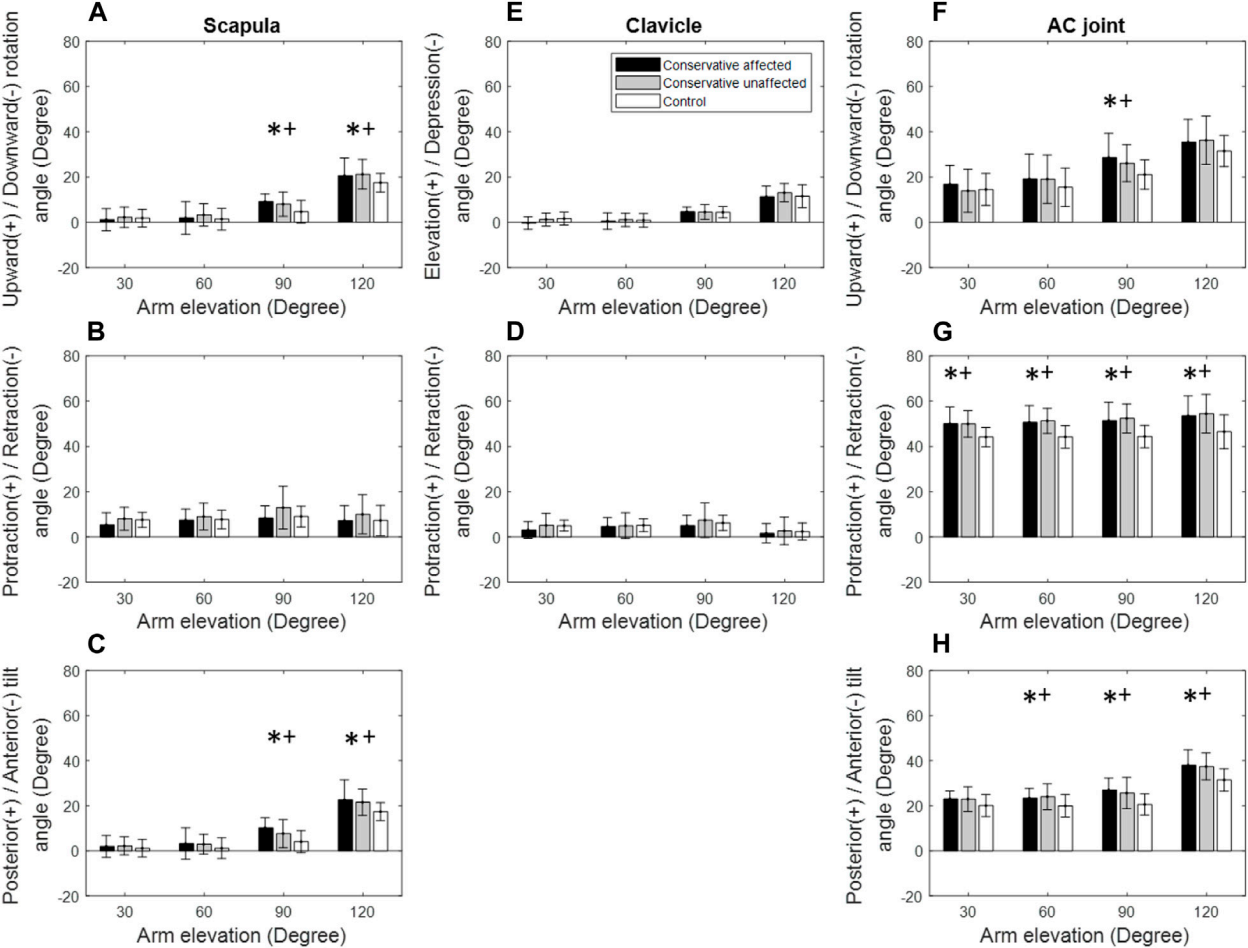
Mean rotational angles of the scapula **(A–C)**, clavicle **(D,E)**, and the acromioclavicular (AC) joint **(F–H)** during sagittal-plane arm elevation for both the patient (black bars: affected side; grey bars: unaffected side) and control (white bars) groups. Significant differences between the affected side and healthy control are indicated by the symbol *, while significant differences between the unaffected side and healthy control are indicated by the symbol +. Standard deviations are shown as error bars.

**FIGURE 6:**
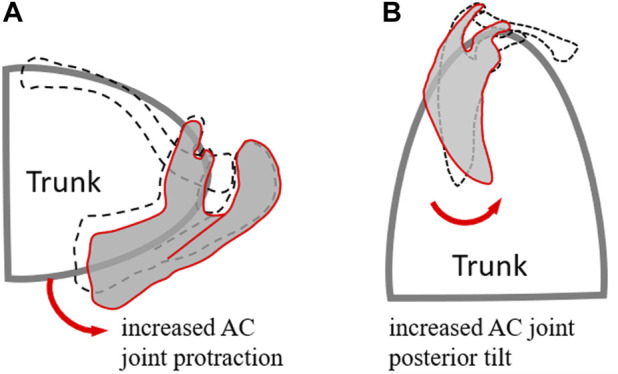
Schematic diagram showing the different kinematics of the scapula and the AC joint between the patient (red lines) and healthy control (black dash lines) groups in the transverse plane **(A)** and sagittal plane **(B)** during frontal-plane arm elevation. Compared to healthy controls, the patient group showed increased scapular protraction and posterior tilt while maintaining unaltered scapular rotation.

During sagittal-plane arm elevation on the affected side, the Patient group showed significantly greater upward rotation and posterior tilt of the scapula at 90° and 120° of elevation than the Control group (Figure 5). The Patient group also showed significantly greater AC protraction at 30°–120° of elevation and posterior tilt at 60°–120° of elevation (Figure 5). Similar findings were found for the unaffected side (Figures 5, 6).

## Discussion

The current study used computerised optical motion analysis to quantify the effects of conservative management on the 3D poses of the shoulder bones during multi-plane elevations in patients with mid-shaft clavicle fractures. By the time of the motion experiment, the patients had full clavicle union and recovered general shoulder function, with good to excellent subjective outcome measures. Normal shoulder kinematics was also recovered for arm elevations up to 60° in all three tested planes despite residual angular deformities of the clavicle. For elevations beyond 60°, normal clavicle kinematics but significantly increased scapular posterior tilt relative to the trunk was observed, leading to significantly increased clavicular protraction and posterior tilt relative to the scapula (i.e., AC joint). In the sagittal plane, additional changes of increased scapular upward rotations at 90° and 120° elevations were found. The arm elevation ranges with increased clavicular protraction and posterior tilt at the AC joint were minimal in the frontal plane and maximal in the sagittal plane. Similar kinematic alterations were observed on the unaffected side, indicating a tendency for symmetrical bilateral adaptation. The results suggest that despite residual clavicular malunion, the patients showed normal shoulder kinematics for arm elevations up to 60° in all three elevation planes. Still, bilateral compensatory kinematic changes of the scapula and AC joint were needed for arm elevations beyond 60° in all three planes. After bone healing, rehabilitative training on both sides may be required to improve shoulder complex movement control for higher humeral elevations. The study findings could help inform treatment decisions and improve clinical outcomes.

During frontal-plane arm elevation, the Patient group showed normal shoulder kinematics on the affected side for arm elevations up to 60°, suggesting that despite residual angular deformities of the clavicle, normal shoulder kinematics were recovered within moderate arm elevation ranges (Figure 1). This phenomenon may also be related to the scapular-humeral rhythm that is less affected by the scapular and clavicle motions during the early rage of arm elevation (Fung et al., 2001). For higher arm elevations (90° and 120°), relative to the trunk, normal clavicle kinematics but significantly increased scapular posterior tilt was observed, leading to altered AC joint kinematics with significantly increased scapular protraction and posterior tilt relative to the clavicle (Figure 3). Since the shoulder complex is a kinematic mechanism of the individual bones (Su et al., 2016; Merolla et al., 2019; Li et al., 2020), any morphological deformity of a bone or kinematic changes of a joint can lead to compensatory kinematic changes at other joints or kinematic components (Kibler and Sciascia, 2010; Keshavarz et al., 2017; Merolla et al., 2019). In brief, sagittal and transverse plane secondary kinematic changes in the scapula and AC joint accompanied the main motion components to achieve the required frontal-plane arm elevation.

Kinematic changes within the shoulder complex during scapular-plane elevation were similar to those in the frontal plane (Figures 3, 4). However, those in the sagittal plane were slightly different (Figure 5), reflecting the three-dimensional nature of the shoulder complex. Compared to the frontal and scapular plane elevations, sagittal-plane elevations required additional changes of increased scapular upward rotations at 90° and 120°. Given that the shoulder complex is a kinematic mechanism, it appears reasonable that arm elevations in the sagittal plane tended to rotate the scapula laterally and tilt the scapula posteriorly with normal clavicular kinematics for all test planes of arm elevation. These results suggest that for the desired amount of arm elevation, the AC joint played a critical role in the observed kinematic changes of the scapula, showing increased protraction and posterior tilt relative to the scapula with the greatest magnitudes during sagittal-plane arm elevations and the smallest during frontal-plane elevations (Figures 3–5). The AC and scapular kinematics changes may be related to the previously reported muscular weakness or limitations of joint motion, which may be further amplified with clavicular malunion (Shklar and Dvir, 1995; Mirzatolooei, 2011; Kim et al., 2017). These kinematic changes may lead to long-term negative consequences during repeated daily activities involving shoulder elevations beyond 60°, such as drinking with a cup, wearing glasses and touching the other side of the shoulder (Kibler et al., 2013). Based on the current findings, rehabilitation strategies for patients with mid-shaft clavicle fractures should aim to restore normal shoulder kinematics for arm elevations up to 60°, as conservative management effectively achieved this outcome. For elevations beyond 60°, rehabilitation should restore out-of-elevation-plane kinematic changes of the scapula and clavicle to normalise the AC joint, including exercises to improve the upward scapular rotation. Additionally, exercises to enhance clavicular protraction and posterior tilt relative to the scapula may be beneficial.

Note that the unaffected shoulder complex also showed kinematic deviations and secondary changes similar to those within the affected side (Figure 3), indicating a trend of symmetrical bilateral compensation. A possible explanation is due to bilateral symmetrical muscular controls during arm elevations. Muscular control of the shoulder motion is complex–different muscles are active during various phases of the arm elevations in different planes. For example, the supraspinatus fires during the first 15° of elevation; the deltoid is active up to 90°; the trapezius and serratus anterior are active for arm elevations beyond 90° (Bagg and Forrest, 1988; Ludewig et al., 1996; Borstad and Ludewig, 2002; Ludewig et al., 2009; McCausland et al., 2023). Further studies using electromyography measurements may help provide more insight into the bilateral neuromuscular control of the shoulder complex during arm elevations. Nonetheless, rehabilitative training on both sides, such as muscle strengthening and synergistic balance, may be needed to improve the movement control of the shoulder complex during humeral elevation.

The residual kinematic deviations and compensatory changes of the shoulder complex in the patients treated non-surgically for mid-shaft clavicle fractures were mainly in the sagittal and frontal planes. These results contrast findings from a recent study of patients with internal fixation for mid-shaft clavicle fractures (Hung et al., 2021). During frontal-plane elevations, surgically treated patients had reduced clavicular retraction with compensatory changes in scapular kinematics, primarily in the transverse plane, namely, increased scapular protraction at lower elevation angles and decreased scapular retraction at higher elevation angles. During the sagittal-plane and scapular-plane elevations, the elevation angles with significant scapular kinematic changes were reduced to 60° and 90° without altering the clavicular kinematics. The residual deviations and compensations in the current study involved mainly the kinematic changes at the AC joint, while those in surgically treated patients affected both the AC and sternoclavicular (SC) joints. Such differences may be related to the conditions of the articular geometry of the bones, ranges of motion of the AC and SC joints, muscular attachments on the clavicle, and muscle strength following the treatment (Ledger et al., 2005; Nowak et al., 2005; Zlowodzki et al., 2005; McKee et al., 2006; Rosenberg et al., 2007). Nonetheless, both treatment methods led to bilateral symmetrical kinematic compensations, suggesting that rehabilitation strategies should also consider compensatory kinematic changes on the unaffected side. On the other hand, the current study focused on shoulder kinematics during arm elevations. Future studies will be needed to test whether there would be shoulder kinematic differences between arm elevation and lowering movements.

The current study was the first attempt in the literature to quantify non-invasively the possible residual kinematic deviations of the shoulder during multi-plane elevations in patients with treated mid-shaft clavicle fractures by a standardised protocol that includes shoulder immobilisation with a broad arm sling with a figure-of-eight bandage and physical therapy. For the assessment of the 3D dynamic shoulder skeletal motions during more complex functional activities, medical imaging approaches, such as 3D fluoroscopy (Lin et al., 2014; Lin et al., 2018; Lin et al., 2020), may be used. The current findings could help inform treatment decisions and improve clinical outcomes, including optimising rehabilitation programs to address all the causative factors that can alter the balance of muscles (Kibler and McMullen, 2003) and the inflexibility of the shoulder (Kibler et al., 2012). Considering the three-dimensional nature and mechanism of the shoulder complex, a comprehensive rehabilitation program tailored to the needs of each patient with mid-shaft clavicle fractures could lead to optimal recovery and improved outcomes. Note that the current study utilised a cross-sectional case-control design. The baseline data of the patient group before the clavicle fractures were unavailable. Although we used the data of the unaffected side as a reference, further longitudinal studies are needed to investigate the effects of conservative treatment with shoulder immobilisation and subject-specific rehabilitation training on the 3D skeletal kinematics of both shoulders in patients with unilateral mid-shaft clavicle fractures. On the other hand, despite a mean age difference of about 10 years between the two groups, no statistically significant difference was found, so we did not take the age as a covariate. Further study on other age groups may be needed.

## Conclusions

The current study quantified the residual kinematic deviations of both shoulders during multi-plane elevations in patients recovered from unilateral mid-shaft clavicle fractures treated by a standard treatment protocol that includes shoulder immobilisation with a sling and physical therapy. Despite residual clavicular malunion, the patients showed normal shoulder kinematics for arm elevations up to 60° in all three elevation planes. For elevations beyond 60°, relative to the trunk, normal clavicle kinematics but significantly increased scapular posterior tilt was observed, leading to altered AC joint kinematics with significantly increased scapular protraction and posterior tilt relative to the clavicle. In the sagittal plane, additional changes of increased scapular upward rotations at 90° and 120° elevations were found. Still, bilateral compensatory kinematic changes of the scapula and AC joint were needed for arm elevations beyond 60° in all three planes, suggesting the need for monitoring for any compromised integrated motions of the individual bones following conservative treatment. Rehabilitative training on both sides is also recommended to improve shoulder complex movement control for higher humeral elevations. The current experimental approach may be used for assessing treatment efficacy in patients with other shoulder injuries and treatment.

## Data Availability

The original contributions presented in the study are included in the article/Supplementary Material, further inquiries can be directed to the corresponding author.
